# The burden of hypertension, diabetes mellitus, and cardiovascular risk factors among adult Malawians in HIV care: consequences for integrated services

**DOI:** 10.1186/s12889-016-3916-x

**Published:** 2016-12-12

**Authors:** Oscar H. Divala, Alemayehu Amberbir, Zahra Ismail, Teferi Beyene, Daniela Garone, Colin Pfaff, Victor Singano, Harriet Akello, Martias Joshua, Moffat J. Nyirenda, Alfred Matengeni, Josh Berman, Jane Mallewa, Gift S. Chinomba, Noel Kayange, Theresa J. Allain, Adrienne K. Chan, Sumeet K. Sodhi, Joep J. van Oosterhout

**Affiliations:** 1 Dignitas International, PO Box 1071, Zomba, Malawi; 2 Pirimiti Rural Hospital, Pirimiti, Malawi; 3 Ministry of Health, Zomba Central Hospital, Zomba, Malawi; 4 Department of Medicine, College of Medicine, Blantyre, Malawi; 5 Ministry of Health, District Health Office, Zomba, Malawi; 6 Toronto Western Hospital, University Health Network, Toronto, Canada

**Keywords:** HIV, Hypertension, Diabetes, Malawi, Antiretroviral, Integrated, Cardiovascular, Africa

## Abstract

**Background:**

Hypertension and diabetes prevalence is high in Africans. Data from HIV infected populations are limited, especially from Malawi. Integrating care for chronic non-communicable co-morbidities in well-established HIV services may provide benefit for patients by preventing multiple hospital visits but will increase the burden of care for busy HIV clinics.

**Methods:**

Cross-sectional study of adults (≥18 years) at an urban and a rural HIV clinic in Zomba district, Malawi, during 2014. Hypertension and diabetes were diagnosed according to stringent criteria. Proteinuria, non-fasting lipids and cardio/cerebro-vascular disease (CVD) risk scores (Framingham and World Health Organization/International Society for Hypertension) were determined. The association of patient characteristics with diagnoses of hypertension and diabetes was studied using multivariable analyses. We explored the additional burden of care for integrated drug treatment of hypertension and diabetes in HIV clinics. We defined that burden as patients with diabetes and/or stage II and III hypertension, but not with stage I hypertension unless they had proteinuria, previous stroke or high Framingham CVD risk.

**Results:**

Nine hundred fifty-two patients were enrolled, 71.7% female, median age 43.0 years, 95.9% on antiretroviral therapy (ART), median duration 47.7 months. Rural and urban patients’ characteristics differed substantially. Hypertension prevalence was 23.7% (95%-confidence interval 21.1–26.6; rural 21.0% vs. urban 26.5%; *p* = 0.047), of whom 59.9% had stage I (mild) hypertension. Diabetes prevalence was 4.1% (95%-confidence interval 3.0–5.6) without significant difference between rural and urban settings. Prevalence of proteinuria, elevated total/high-density lipoprotein-cholesterol ratio and high CVD risk score was low. Hypertension diagnosis was associated with increasing age, higher body mass index, presence of proteinuria, being on regimen zidovudine/lamivudine/nevirapine and inversely with World Health Organization clinical stage at ART initiation. Diabetes diagnosis was associated with higher age and being on non-standard first-line or second-line ART regimens.

**Conclusion:**

Among patients in HIV care 26.6% had hypertension and/or diabetes. Close to two-thirds of hypertension diagnoses was stage I and of those few had an indication for antihypertensive pharmacotherapy. According to our criteria, 13.0% of HIV patients in care required drug treatment for hypertension and/or diabetes.

## Background

In a national survey from 2009, high prevalence of hypertension (33%) and diabetes mellitus (6%) was observed in the Malawi general population [1]. Both conditions are likely to become even more common due to population transition effects related to urbanization, life style changes associated with increasing wealth, as well as increasing age of the population [2]. The World Health Organization (WHO) predicts that the burden of hypertension in sub-Saharan Africa will double between 2000 and 2025 [3]. The main complications of hypertension and diabetes mellitus, such as heart failure, stroke, myocardial infarction and renal failure have not been well studied at population level in Malawi, but are common reasons for admissions to medical departments [4]. It has therefore been widely argued that hypertension and diabetes mellitus need to get more public health priority [5]. Both conditions are also common among HIV infected patients. One small study showed a hypertension prevalence of 46% among Blantyre anti-retroviral therapy (ART) patients [6] but further information from Malawi has not been published. HIV and antiretroviral drugs are believed to increase the incidence of diabetes mellitus according to western studies [7], which is compounded by the increased life expectancy of persons living with HIV and a longer mean exposure to ART. The adult Malawian population on ART is very large (>550,000 persons) and will continue to grow as eligibility for ART extends.

Because persons living with HIV are under regular follow up in the health care system, they can benefit from integrated screening and treatment of hypertension and diabetes mellitus at HIV clinics. When planning integration of hypertension and diabetes management into HIV services, the increased burden for busy HIV clinics has to be considered. How large that burden is, will be determined by the prevalence of hypertension and diabetes as well as by the percentage of patients that need pharmacotherapy which requires more frequent and longer clinic visits. For persons with mild hypertension, who do not have cardio/cerebro-vascular disease (CVD) or target organ damage and have low CVD risk, antihypertensive drug treatment may not be indicated.

We conducted a study in two HIV clinics in Zomba district, Malawi to estimate the prevalence of hypertension, diabetes mellitus and CVD risk factors among adult Malawians in HIV care and to explore the burden of patients in need of integrated pharmacotherapy.

## Methods

### Study setting

A cross-sectional study was carried out at the HIV clinics of Zomba Central Hospital (urban setting, around 7000 patients) and Pirimiti Rural Hospital (rural setting, around 4000 patients). Both clinics are located in Zomba district, southern Malawi, where HIV prevalence is around 14.5% in the 15–49 year age group [8]. After the study we started implementation of integrated HIV-hypertension-diabetes treatment at one of the clinics and will report this experience elsewhere.

### Participants and data collection

Consecutive patients aged ≥18 years accessing HIV care were included in the study. Participants were interviewed by trained research assistants using a structured questionnaire in the local language or English, according to patients’ preference. Household size was defined as number of people aged ≥18 years living in the participant’s household. Self-reported physical activity was categorized as vigorously-, moderately- and in-active, based on the STEPS questionnaire, developed by WHO for non-communicable disease surveillance [1]. In this methodology, inadequate fruit and vegetable consumption is defined as eating less than 5 servings of fruit and/or vegetables per day. Because virtually all (>99.5%) participants had an inadequate diet using these criteria, we used the mean number of vegetable servings per day as a variable in our analyses. HIV-related information was extracted from standard clinic registers and master cards. Physical measurements were performed by study nurses. Participants were asked to rest on a chair for 15 min before three digital blood pressure measurements (Rossmax® AW 150f, Jena, Germany) were taken, three minutes apart. Non-fasting capillary blood samples were collected for all biochemical tests. Blood glucose was measured with the point-of-care SD CHECK™ GOLD Blood Glucose Meter (SD Biosensor, Inc., GyeongGi-do, Republic of Korea). A follow up fasting blood glucose was collected if the blood glucose level was >200 mg/dl. Blood lipid levels were determined using the point-of-care CardioChek**®** PA (Polymer Technology Systems, Inc., Indianapolis, USA) in a sub-sample of the first 277 enrolled patients at each clinic. Urine protein was measured with SD UroColor^TM^10 urine dipsticks that provide a semi-quantitative result (Standard Diagnostics, Inc., Kyonggi-do, Republic of Korea); negative and trace results were considered as normal.

### Clinical definitions

Hypertension was defined as a systolic blood pressure (SBP) of ≥140 mmHg and/or diastolic blood pressure (DBP) ≥90 mmHg of the mean of the first 3 BP measurements and confirmed at two follow-up visits within two weeks. Hypertension was categorized as follows: stage I (mild) hypertension SBP 140–159 mmHg and/or DBP 90–99 mmHg, stage II (moderate) SBP 160–179 mmHg and/or DBP 100–109 mmHg and stage III (severe) SBP ≥180 mmHg and/or DBP ≥110 mmHg. Patients on anti-hypertensive treatment were regarded as having hypertension irrespective of blood pressure readings and were not staged. Body mass index (BMI) was categorized according to WHO thresholds [9]. Central obesity was defined as waist-hip ratio (waist circumference/hip circumference) of ≥0.94 for men and ≥0.80 for women [10]. Diabetes mellitus was diagnosed if an elevated blood glucose level was recorded on two separate occasions: >126 mg/dL if the patient was fasting and >200 mg/dL if not. Participants already on antidiabetic drugs were regarded as diabetics irrespective of their blood glucose level. Total cholesterol (TC) was elevated if >5.2 mmol/L. High-density lipoprotein cholesterol (HDL-c) was decreased if <1.1 mmol/L. Triglyceride (TG) upper level of normal was 2.9 mmol/L. The TC/HDL-c ratio was used as an indicator of increased CVD risk and considered elevated if >5. Framingham risk score (FRS) and WHO/International Society for Hypertension (WHO/ISH) risk scores were calculated for all patients where possible [11, 12] We used the mean of the 3 blood pressure measurements of the first visit in both risk scores. FRS includes sex, age (≥20 years), current smoking status, total cholesterol, HDL-cholesterol, anti-hypertensive drug use and systolic blood pressure. Two WHO/ISH risk scores are based on prediction charts for the African region [12]. One includes sex, age (≥40 years), smoking status, systolic BP and diabetes status, the second also includes TC. Since the WHO/ISH risk prediction charts exclude those aged <40, these scores could not be calculated for 573 patients.

### Statistical analysis

The sample size was based on the expected prevalence of hypertension from a previous study in Malawi (ref 6), a desired precision of 5%, a 95% confidence interval and adding 25% for non-response; 471 participants were thus required per clinic (i.e. 942 in total). Data were entered in an Access database, cleaned, coded and merged ready for analysis using Stata 13 (Statacorp, College Station Texas, USA). We described patients’ demographic and clinical characteristics and cardiovascular risk factors separately for urban and rural clinics. Point-prevalence of hypertension, diabetes, hyperlipidemia, proteinuria and high CVD risk scores is reported with 95%-confidence intervals (95%-CI). Univariate and multivariable logistic regression analyses were performed to determine independent risk factors for hypertension and diabetes mellitus. Age, gender, study site and educational status were kept in the model as *a priori* confounder regardless of degree of association in univariate analysis. Proteinuria was omitted from the diabetes final model due to insufficient numbers. The significance of the association between patients’ characteristics and outcomes in the models was assessed using a likelihood ratio test. *P-value* for trend is reported as appropriate. To estimate the burden of drug treatment for hypertension and diabetes in the HIV population in care, we included all patients with diabetes diagnosis, and those with stage II and III hypertension. We added patients with stage I hypertension who had coexisting proteinuria, self-reported previous stroke, and/or a high FRS. The total number with high FRS was determined by extrapolation using the percentage found among patients with stage I hypertension in whom the score could be determined, i.e. those with lipid measurements. At each clinic, we compared routinely collected patients characteristics of the overall clinic population aged ≥18 years to those of the study population, to assess if the study sample was representative of all HIV patients in care.

## Results

### Demographic, life-style and clinical characteristics

Between July and October 2014, we enrolled 952 patients, 480 in the urban and 472 in the rural clinic. The median age was 43 years and 683 (71.7%) were female. Urban patients had higher education levels, were more frequently employed, less physically active, ate more vegetables and fruits, used less smokeless tobacco, were more often on ART, had a longer duration of HIV infection and duration on ART, less advanced clinical HIV disease at the start of ART, were less commonly on the standardized first-line ART regimen and had a higher BMI than rural patients. Overall prevalence of smoking and alcohol usage was low (Table 1). When comparing the study populations with the overall adult patient populations at each clinic, we found that the study populations were significantly older, longer on ART, and less often on the standardized first-line regimen, while only in the urban clinic the percentage of females was higher than in the overall patient population (data not shown).

**Table 1:** Characteristics of patients in HIV care in a rural and an urban clinic, Zomba District, Malawi

Characteristics	Total	Rural	Urban	*P*-value
	n (%)	n (%)	n (%)	
*Number of patients*	952	472 (49.6)	480 (50.4)	
*Gender*				
Female	683 (71.7)	334 (70.8)	349 (72.7)	0.505
Male	269 (28.3)	138 (29.2)	131 (27.3	
*Age in years (N = 952)*				0.250
18–34	210 (22.1)	111 (23.5)	99 (20.6)	
35–44	361 (37.9)	182 (38.6)	179 (37.3)	
45–54	238 (25.0)	105 (22.3)	133 (27.7)	
≥ 55	143 (15.0)	74 (15.7)	69 (14.4)	
*Mean age (SD) in years*	43.0 (10.2)	42.7 (10.6)	43.2 (9.8)	0.471
*Educational status*				
None	204 (21.5)	143 (30.3)	61 (12.7)	<0.001
Standard 1–8	576 (60.6)	283 (60.0)	293 (61.2)	
Secondary & above	171 (18.0)	46 (9.8)	125 (26.1)	
*Household size mean (SD)*	2.58 (1.26)	2.55 (1.17)	2.62 (1.35)	0.401
*Employment status*				
Employed	112 (11.8)	17 (3.6)	95 (19.8)	<0.001
Unemployed	255 (26.8)	38 (8.1)	217 (45.2)	
Self Employed	585 (61.5)	417 (88.4)	168 (35.0)	
*Physical activity*				
Vigorously active	854 (89.7)	458 (97.0)	396 (82.5)	<0.001
Moderately active	90 (9.5)	11 (2.3)	79 (16.5)	
Inactive individuals	8 (0.8)	3 (0.6)	5 (1.0)	
*Dietary history*				
Mean days of fruit consumption/wk (SD)	2.63 (2.06)	2.31 (1.87)	2.98 (2.20)	<0.001
Mean servings of fruit/day (SD)	1.25 (0.76)	1.20 (0.74)	1.31 (0.79)	0.028
Mean days of vegetables consumption/wk (SD)	5.13 (1.95)	4.51 (1.88)	5.73 (1.83)	<0.001
Mean servings of vegetables/day (SD)	1.81 (0.49)	1.77 (0.50)	1.85 (0.47)	0.006
*Tobacco*				
Ever smoked tobacco	117 (12.3)	50 (10.6)	67 (14.0)	0.114
Current tobacco smokers	34 (3.6)	19 (4.3)	15 (3.1)	0.454
Daily tobacco smoker	25 (2.6)	15 (3.2)	10 (2.1)	0.291
Current smokeless tobacco users	21 (2.2)	15 (3.2)	6 (1.3)	0.041
*Alcohol*				
Used alcohol in the past 30 days (current drinker)	61 (6.4)	33 (7.0)	28 (5.8)	0.466
Used alcohol in the past 12 months	97 (10.2)	56 (11.9)	41 (8.5)	0.090
*Self-report of previous stroke*				
Yes	41 (4.3)	26 (5.5)	15 (3.1)	0.070
No	909 (95.7)	445 (94.5)	464 (96.9)	
*Self-report of previous myocardial infarction*				
Yes	211 (22.2)	113 (24.0)	98 (20.5)	0.190
No	739 (77.8)	358 (76.0)	381 (79.5)	
*ART Status*				
On ART	913 (95.9)	443 (93.9)	470 (97.9)	0.002
Pre-ART	39 (4.1)	29 (6.1)	10 (2.1)	
*Mean HIV infection duration (SD), months*	55.4 (31.7)	49.7 (32.2)	61.0 (30.2)	<0.001
*Mean ART duration (SD), months*	47.7 (29.3)	42.1 (29.0)	53.0 (28.7)	<0.001
*CD4 count at ART initiation, mean (SD) (n = 632)*	217.5 (114.6)	216.9 (121.0)	218.1 (108.2)	0.898
*WHO stage at ART initiation*				
WHO Stage 1	275 (30.1)	171 (38.6)	104 (22.1)	<0.001
WHO Stage 2	306 (33.5)	139 (31.4)	167 (35.5)	
WHO Stage 3	270 (29.5)	110 (24.8)	160 (34.0)	
WHO Stage 4	63 (6.9)	23 (5.2)	40 (8.5)	
*Current ART Regimen*				
2A^a^	107 (11.7)	33 (7.5)	74 (15.7)	<0.001
5A^b^	775 (84.9)	397 (89.6)	378 (80.4)	
Other	31 (3.4)	13 (2.9)	18 (3.8)	
*Mean BMI (SD), kg/m2*	21.9 (3.4)	21.3 (3.2)	22.6 (3.6)	<0.001
*BMI categories, n (%)*				
Under weight (<18.5 kg/m^2^)	102 (10.7)	65 (13.8)	37 (7.7)	<0.001
Normal (18.5–25 kg/m^2^)	682 (71.6)	349 (73.9)	333 (69.4)	
Over weight (>25–30 kg/m^2^)	132 (13.9)	45 (9.5)	87 (18.1)	
Obesity (>30 kg/m^2^)	36 (3.8)	13 (2.8)	23 (4.8)	
*Waist to hip ratio, n (%)*				
High	249 (26.9)	122 (27.1)	127 (26.6)	0.867
Normal	678 (73.1)	328 (72.9)	350 (73.4)	
*Proteinuria*				
Normal (negative or trace)	928 (97.9)	470 (99.6)	458 (97.9)	0.001
1+	13 (1.4)	2 (0.4)	11 (2.3)	
2+	7 (0.7)	0	7 (1.5)	

*SD* standard deviation, *BMI* body mass index

^a^2A, generic single tablet formulation of zidovudine, lamivudine, nevirapine

^b^5A, generic single tablet formulation of tenofovir, lamivudine, efavirenz

### Hypertension and diabetes prevalence and risk factors

Hypertension was diagnosed in 226 patients, resulting in an overall prevalence of 23.7% (95%-CI 21.1–26.6), which included 29 patients (12.8%) already on pharmacotherapy and 166 (73.5%) newly diagnosed cases. Of patients with untreated hypertension (*n* = 197; 87.2%), 118 (59.9%) had stage I hypertension, 61 (31.0%) stage II and 18 (9.1%) stage III. Hypertension was more common among urban patients (Table 2). Variables that were independently associated with a diagnosis of hypertension were increasing age, obesity, current ART regimen zidovudine/lamivudine/nevirapine and presence of proteinuria, while an inverse independent association was observed with WHO clinical stage at ART initiation (Table 3). We diagnosed 39 patients with diabetes mellitus, of whom 10 (25.6%) were already on pharmacotherapy. The overall prevalence of diabetes mellitus was 4.1% (95%–CI 3.0–5.6), similar in urban and rural patients (Table 2). A diagnosis of diabetes mellitus was independently associated with current ART regimen other than tenofovir/lamivudine/efavirenz and zidovudine/lamivudine/nevirapine, and with increasing age (Table 3).

**Table 2:** Prevalence of hypertension, diabetes mellitus, dyslipidaemias, elevated cardio/cerebro-vascular disease risk and proteinuria among patients at a rural and an urban HIV clinic in Zomba District, Malawi

Outcomes	Overall		Rural		Urban		*p*-value
	n/N (%)	95% CI	n/N (%)	95% CI	n/N (%)	95% CI	
Hypertension	226/952 (23.7%)	21.1–26.6	99/472 (21.0)	17.5–24.9	127/480 (26.5)	22.7–30.6	0.047
Diabetes mellitus	39/952 (4.1%)	3.0–5.6	16/472 (3.4)	2.1–5.5	23/480 (4.8)	3.2–7.1	0.275
Hypertension + diabetes mellitus	11/952 (1.2%)	0.6–2.1	3/472 (0.6)	0.2–1.9	8/480 (1.7)	0.8–3.3	0.137
Raised total cholesterol	86/554 (15.5%)	12.7–18.8	44/277 (15.9)	10.0–20.7	42/277 (15.2)	11.4–90.9	0.814
Decreased HDL cholesterol	88/554 (15.9%)	13.1–19.2	46/277 (16.6)	12.7–21.5	42/277 (15.2)	11.4–19.9	0.642
Raised total/HDL cholesterol ratio	21/554 (3.8%)	2.5–5.6	11/277 (4.0)	2.2–7.1	10/277 (3.6)	1.9–6.6	0.842
Hypertriglyceridemia	159/554 (28.7%)	25.1–32.6	87/277 (31.4)	26.2–37.1	72/277 (26.0)	21.1–31.5	0.159
Framingham score, risk >20%^a^	13/551 (2.4%)	1.4–4.0	8/277 (2.9)	1.4–5.7	5/274 (1.8)	0.8–4.3	0.411
WHO/ISH score, risk >20%^a^	13/379 (3.4%)	2.0–5.8	6/180 (3.3)	1.5–7.3	7/199 (3.5)	1.8–7.2	0.922
Proteinuria^b^	20/948 (2.1%)	1.4–3.2	2/472 (0.4%)	0.1–1.7	18/476 (3.8%)	2.4–6.0	0.001

*n* numerator, *d* denominator, *HDL* high-density lipoprotein, *WHO* World Health Organization, *ISH* International Society for Hypertension

^a^Indicating the risk of experiencing a fatal or non-fatal myocardial infarction or stroke in the next 10 years

^b^Grade 1+ and 2+ proteinuria as determined by dipstick urine testing

**Table 3:** Factors associated with hypertension and diabetes mellitus among patients at a rural and an urban clinic in Zomba District

	Hypertension				Diabetes Mellitus			
Characteristic	n (%) HTN	Crude OR (95% CI)	Adjusted OR (95% CI)	*P*-value*	n (%) DM	Crude OR (95% CI)	Adjusted OR (95% CI)	*P*-value*
*Study site (N = 952)*								
Rural	99 (21.0)	1	1	0.66	16 (3.4)	1	1	0.40
Urban	127 (26.5)	**1.36 (1.00, 1.83)**	1.09 (0.74, 1.60)		23 (4.8)	1.43 (0.75, 2.75)	1.38 (0.65, 2.92)	
*Gender (N = 952)*								
Female	160 (23.4)	1	1	0.94	29 (4.3)	1	1	0.32
Male	66 (24.5)	1.06 (0.76–1.48)	0.98 (0.65, 1.49)		10 (3.7)	0.87 (0.42–1.81)	0.63 (0.26, 1.55)	
*Age in years (N = 952)*				<0.01*				<0.12*
				*<0.01* ^*¥*^				*0.02* ^*¥*^
18–34	22 (10.5)	1	1		5 (2.4)	1	1	
35–44	47 (13.0)	1.28 (0.75, 2.19)	1.09 (0.61, 1.96)		13 (3.6)	1.53 (0.54, 4.36)	1.51 (0.47, 4.91)	
45–54	87 (36.6)	**4.92 (2.94, 8.24)**	**4.50 (2.55, 7.95)**		11 (4.6)	1.99 (0.68, 5.81)	2.16 (0.64, 7.27)	
≥ 55	70 (49.0)	**8.19 (4.72, 14.20)**	**7.46 (4.02, 13.84)**		10 (7.0)	3.08 (1.03, 9.22)	**3.91 (1.12, 13.62)**	
*Education level (N = 952)*				0.37				0.85
None	62 (30.4)	1	1		8 (3.9)	1	1	
Standard 1–8	122 (21.2)	**0.62 (0.43–0.88)**	0.73 (0.48, 1.13)		25 (4.3)	1.11 (0.49–2.51)	1.23 (0.51, 2.98)	
Secondary & above	42 (24.6)	0.75 (0.47–1.18)	0.83 (0.46, 1.49)		6 (3.5)	0.89 (0.30–2.62)	1.01 (0.31, 3.30)	
*Household size (N = 951)*								
≤ 3	180 (22.9)	1			35 (4.5)	1		
> 3	46 (27.9)	1.30 (0.89, 1.90)			4 (2.4)	0.53 (0.19, 1.52)		
*Employment status (N = 952)*								
Employed	30 (26.8)	1			6 (5.4)	1		
Unemployed	58 (22.8)	0.80 (0.48–1.34)			9 (3.5)	0.65 (0.22–1.86)		
Self Employed	138 (23.6)	0.84 (0.53–1.34)			24 (4.1)	0.76 (0.30–1.89)		
*Physical activity (N = 952)*								0.51
Less active	33 (33.7)	1	1	0.56	6 (6.1)	1	1	
Active	193 (22.6)	0.58 (0.37–0.90)	0.85 (0.49, 1.48)		33 (3.9)	0.62 (0.25, 1.51)	0.72 (0.27, 1.91)	
*Fruit servings/ day (N = 906)*								
≤ 1	147 (22.8)	1			30 (4.7)	1		
> 1	64 (24.5)	1.10 (0.79. 1.54)			9 (3.5)	0.73 (0.34, 1.56)		
*Vegetables servings/day (N = 951)*								
≤ 1	45 (22.8)	1			10 (5.1)	1		
> 1	181 (24,0)	1.07 (0.74, 1.55)			29 (3.9)	0.75 (0.36, 1.56)		
*Current smokers (N = 952)*								
No	219 (23.9)	1			36 (3.9)	1		
Yes	7 (20.6)	0.83 (0.36–1.93)			3 (8.8)	2.37 (0.69–8.12)		
*Alcohol use in the past 12 months (N = 952)*								
No	206 (24.1)	1			37 (4.3)	1		
Yes	20 (20.6)	0.82 (0.49–1.37)			2 (2.1)	0.47 (0.11–1.96)		
*ART Status (N = 952)*								
On ART	216 (23.7)	1			38 (4.2)	1		
Pre-ART	10 (25.6)	1.11 (0.53–2.32)			1 (2.6)	0.61 (0.08–4.53)		
*ART duration, months (N = 913)*								
≤ 24	40 (17.5)	1	1	0.77	5 (2.2)	1	1	0.18
> 24	176 (25.7)	**1.64 (1.12, 2.40)**	1.07 (0.66, 1.73)		33 (4.8)	2.27 (0.88, 5.89)	2.17 (0.70, 6.70)	
*CD4 count at ART initiation (N = 632)*								
≤ 250	104 (25.2)	1			17 (4.1)	1		
> 250	47 (21.4)	0.80 (0.54, 1.19)			9 (4.1)	0.99 (0.43, 2.26)		
*WHO stage at ART initiation (N = 914)*				0.01				
WHO Stage 1	49 (17.8)	1	1		8 (2.9)	1	1	0.99
WHO Stage 2	81 (26.5)	**1.66 (1.11–2.48)**	1.23 (0.76, 1.99)		13 (4.3)	1.48 (0.60–3.63)	1.10 (0.42, 2.85)	
WHO Stage 3	77 (28.5)	**1.84 (1.22–2.76)**	1.13 (0.68, 1.87)		14 (5.2)	1.83 (0.75–4.42)	1.01 (0.38, 2.68)	
WHO Stage 4	10 (15.9)	0.87 (0.41–1.83)	**0.31 (0.13, 0.76)**		3 (4.8)	1.67 (0.43–6.48)	1.11 (0.27, 4.62)	
*Current ART regimen (N = 913)*				<0.01				
5A	157 (20.3)	1	1		30 (3.9)	1	1	0.01
2A	49 (45.8)	**3.33 (2.19, 5.05)**	**2.80 (1.71, 4.61)**		3 (2.8)	0.72 (0.21, 2.39)	0.46 (0.13, 1.58)	
Other	10 (32.3)	1.87 (0.86–4.06)	1.96 (0.82, 4.71)		5 (16.1)	**4.78 (1.71–13.30)**	**4.75 (1.60, 14.11)**	
*BMI categories (N = 952)*				<0.01*				0.73*
				*<0.01* ^*¥*^				*0.37* ^*¥*^
Underweight	145 (21.3)	1	1		25 (3.7)	1	1	
Normal	21 (20.6)	0.96 (0.57, 1.61)	0.76 (0.43, 1.35)		6 (5.9)	1.64 (0.66, 4.11)	1.65 (0.63, 4.33)	
Overweight	40 (30.0)	**1.61 (1.06–2.44)**	1.47 (0.90, 2.41)		6 (4.6)	1.25 (0.50–3.11)	1.33 (0.51, 3.46)	
Obesity	20 (55.6)	**4.63 (2.34–9.16)**	**3.67 (1.56, 8.64)**		2 (5.6)	1.54 (0.35–6.80)	1.55 (0.33, 7.39)	
*Waist-to-hip ratio (N = 927)*				0.56				0.85
Normal	157 (23.2)	1	1		26 (3.8)	1	1	
Elevated	67 (26.9)	1.22 (0.88–1.70)	1.13 (0.75, 1.71)		12 (4.8)	1.27 (0.63–2.56)	1.08 (0.50, 2.31)	
Proteinuria (N = 948)**				0.08*				
				0.04^¥^				
Normal	218 (23.5)	1	1		38 (4.1)	1		
1+	2 (15.4)	0.59 (0.13–2.69)	0.92 (0.16, 5.25)		0 (0.0)	-		
2+	5 (71.4)	**8.14 (1.57–42.26)**	**8.25 (1.25, 54.44)**		1 (14.3)	3.90 (0.46–33.24)		

Age, sex, study site and educational status kept in the model as *a priori* confounder regardless of degree of association in univariable analysis

*HTN* hypertension, *DM* diabetes mellitus, *OR* odds ratio, *CI* confidence interval, *ART* antiretroviral therapy, *WHO* World Health Organization, *BMI* body mass index

*LHR test

^¥^Ptrend = *p*-value for trend

** Proteinuria omitted from diabetes final model due to insufficient numbers

Statistically significant findings are indicated in bold font

### Proteinuria, dyslipidaemia and CVD risk scores

Proteinuria prevalence was 2.1% and significantly higher in urban patients (Table 2). We screened the first 277 patients enrolled in each clinic for dyslipidaemia. Prevalence of elevated total cholesterol was 15.5%, decreased HDL-cholesterol 15.9%, elevated triglycerides 28.7% and elevated total cholesterol/HDL-cholesterol ratio 3.8% (Table 2). There was no significant difference in prevalence of dyslipidaemias between rural and urban patients. We were unable to determine CVD risk scores in many patients due to age restrictions and our limitation of having lipids from 554 patients. We report the prevalence of a combined risk of >20% to experience a fatal or non-fatal myocardial infarction or stroke in the next 10 years. This prevalence was low among those tested: using the WHO/ISH score excluding total cholesterol (*n* = 379), prevalence was 3.4% and with the same score including total cholesterol (*n* = 187) was 2.1%; using the FRS (*n* = 551) prevalence was 2.4% (Table 2).

### Expected burden of integrated pharmacological treatment

Of patients with stage I hypertension, 86.4% had a low risk FRS in combination with absence of diabetes, proteinuria, and previous stroke. As they would not have an indication for drug treatment, overall 10.0% of the HIV patients in care were in need of antihypertensive pharmacotherapy. The total burden of pharmacological treatment for hypertension and/or diabetes among HIV patients in care, with its associated longer and more frequent clinic visits, was 13.0%. This included all patients with diabetes diagnosis, stage II and III hypertension and those with stage I hypertension and proteinuria, previous stroke, and/or high risk FRS.

## Discussion

Among nearly 1000 adult Malawians in HIV care, the vast majority on ART, we found that the prevalence of hypertension was 23.7%. Hypertension was previously undiagnosed in three quarters, significantly more common at the urban clinic (absolute difference 5.5%) and in nearly two-thirds it was mild (stage I). Diabetes mellitus was present in 4.8% of hypertensive patients. An earlier study of urban ART patients from Malawi found a much higher hypertension prevalence (46%), which may be explained by the less stringent blood pressure measurement methodology than in our study and a small sample size [6]. Several other studies from the region found more comparable hypertension prevalence among ART patients. In two urban studies from Tanzania, prevalence was 28.7% [13] and 26.2% [14] and in a large peri-urban cohort in Uganda 27.9% [15]. In a referral hospital in Cameroon 38% of ART patients had hypertension [16]. In a recent, large (n >25,000 adults; HIV prevalence 10%), population-based study in a rural and an urban area in Malawi, overall hypertension prevalence was 13–16% and diabetes prevalence 1.8–2.6%, and these rates were slightly lower in HIV infected persons [17].

We found that increasing age was independently associated with hypertension, while there was a borderline association with higher BMI. Ageing and obesity are well established risk factors for hypertension [3] but our observation that being on zidovudine-lamivudine-nevirapine was associated with hypertension is surprising. To our knowledge, there is no literature linking either zidovudine or nevirapine to hypertension and this finding therefore needs confirmation. Proteinuria was associated with hypertension, but prevalence was low in our population, possibly related to the fairly low mean age, low rates of moderate/severe hypertension and diabetes and the fact that nearly all patients were on ART. The prevalence of diabetes in our population (4.1%) was slightly lower than observed in the general Malawi population (5.6%), with no significant difference between rural and urban patients. One quarter was previously diagnosed and on medication. There was an expected association of diabetes with higher age. The only other independent risk factor for diabetes was being on an “other” ART regimen. This category included protease inhibitors and stavudine, drugs known to increase the risk of diabetes [17, 18]. Studies among sub-Saharan African ART patients showed fairly similar diabetes prevalences: 1.2% raised blood glucose in Blantyre, Malawi [6], 3.7% diabetes in Tanzanians [14] and 4.1% in South Africans [19]. A small study from Tanzania found a very high diabetes prevalence of 18% in a population of whom 12% used protease inhibitors [20].

As the epidemic of non-communicable diseases in sub-Saharan Africa gains more recognition, policy makers can take the opportunity to integrate services for hypertension and diabetes mellitus into the well-established HIV care infrastructure. Appropriate diagnostic tools for both conditions are inexpensive and readily available in the local setting. Our study shows that screening of HIV patients in care can address a large unmet need, as three-quarters of cases of hypertension and diabetes were not diagnosed previously. Integrated HIV-hypertension-diabetes care will prevent patients making multiple visits to different clinics which often generates high costs and may affect adherence [21, 22], which is crucial for the treatment of all three conditions. Our observations provide insight into the extra burden of care for HIV clinics associated with integrated treatment for hypertension and diabetes. In Malawi, where severe shortages of health care staff prevail even at relatively advantaged HIV clinics, it will be difficult to absorb drug treatment for all the HIV patients in care with hypertension and/or diabetes, as their combined prevalence was high (26.6%). Priority can be given to those most in need and most likely to benefit from drug treatment. Unless CVD, diabetes, target organ damage or high CVD risk co-exists, a large group of HIV patients with stage I hypertension may experience limited, if any benefit from antihypertensive pharmacotherapy [23, 24]. Excluding low risk patients with mild hypertension from drug treatment would reduce the total burden of integrated pharmacotherapy and it’s associated more frequent and longer clinic visits by 50% (from 26.6 to 13.0%).

Strengths of our study include the use of robust definitions of hypertension and diabetes, avoiding overestimation of disease burden as a result of single session blood pressure measurements and one-off elevated blood glucose tests. We combined a large sample size with thorough analysis of CVD risk factors. We believe that our findings can be extrapolated to HIV clinics in many parts of Malawi and sub-Saharan Africa, where patients are mostly on the same standardized ART regimens and have comparable age and gender distributions. Because the rural and urban populations differed considerably it is useful that findings by clinic are valid, sample sizes ensuring adequate power at each clinic. Limitations of our study should also be considered. Due to the cross-sectional nature of the study we were unable to diagnose cases that may have arisen on prospective screening among patients with borderline results. Further, we found that study populations were older, longer of ART and had more female preponderance than the general clinic populations. This could be due to the fact that during the study period certain patients did not visit the clinic, and/or reflect unsuccessful consecutive enrolment of attending patients. The higher age in the study population may have biased our results towards overestimation of the prevalence of hypertension and diabetes. The data concerning self-reported previous stroke could not be verified with formal medical records which may have affected accuracy. We used FRS and WHO/IHS scores for CVD risk estimation. Their usefulness in sub-Saharan Africans is uncertain, since they have not been validated with clinical endpoints. Despite attempts to recalibrate the FRS for HIV infection [25] it remains equally unclear whether and to what degree HIV infection and being on ART increase the priority to treat hypertension compared to classic CVD risk factors. Samples for blood lipids were taken in the non-fasting state, but differences with fasting results are generally small and impact on making clinical decisions concerning CVD prevention is believed to be very limited [26]. We did not determine serum creatinine levels, reflecting a widespread inability of estimating renal function in Malawian laboratories, nor did we assess hypertensive retinopathy and left ventricular hypertrophy and therefore we may have underestimated the proportion of stage I hypertension patients with target organ damage.

## Conclusion

Among adult Malawian HIV patients in care we found high combined prevalence of mostly newly diagnosed hypertension and diabetes. Integrated HIV-hypertension-diabetes care may be individually beneficial but increases the burden of care for busy HIV clinics. Excluding patients with mild hypertension and low CVD risk from drug treatment would half the overall burden of HIV patients in need of integrated pharmacotherapy for diabetes and/or hypertension. More research on CVD risk in HIV infected Africans is urgently needed to generate evidence that is based on clinical endpoints. The individual benefits of integrated care for hypertension and diabetes at HIV clinics needs further study as well as the optimal model of care.

## Abbreviations

ART: Antiretroviral therapy
CVD: Cerebro/cardio-vascular diseases
DBP: Diastolic blood pressure
FRS: Framingham risk score
HDL: High-density lipoprotein
ISH: International Society for Hypertension
SBP: Systolic blood pressure
STEPS: STEP-wise approach to non-communicable disease surveillance by WHO
TC: Total cholesterol
TG: Triglycerides
WHO: World Health Organization.
